# Evaluation of a Neck Mass in a Six-Month-Old Infant: A Case Report

**DOI:** 10.7759/cureus.44233

**Published:** 2023-08-28

**Authors:** Renee Bruce, Mounika Katyayani, Nicholas Pereira

**Affiliations:** 1 Clinical Sciences, Saint James School of Medicine, Kingstown, VCT; 2 Pediatrics, Community Medical Center, Srikakulam, IND; 3 Pediatrics, South Texas Health System Children’s, Edinburg, USA

**Keywords:** methicillin-resistant staphylococcus aureus, neoplasia, acute bacterial cervical lymphadenitis, diagnostic challenge, pediatric neck masses

## Abstract

Pediatric neck masses are one of the commonly encountered problems in clinical practice. They comprise a wide spectrum of congenital to acquired lesions manifesting in early or late childhood. They often pose a diagnostic challenge to the physician. We hereby present a case report of a six-month-old boy with a left-sided neck mass. Findings of a detailed workup were consistent with acute bacterial cervical lymphadenitis, ruling out neoplasia. The child showed significant improvement after including extended coverage of antibiotic therapy for methicillin-resistant *Staphylococcus aureus* (MRSA) and was put under follow-up.

## Introduction

Pediatric neck masses are one of the most commonly encountered problems in otorhinolaryngological practice. They broadly fit into two different categories: congenital and acquired [1]. Branchial cleft cysts, thyroglossal duct cysts, cystic hygromas, laryngoceles, dermoid cysts, teratomas, and hemangiomas fall under the congenital category [1,2]. Acquired neck masses include infectious and neoplastic causes. Benign neoplastic lesions include lipomas, fibromas, and neurofibromas. Malignant lesions occurring in the neck include lymphomas, rhabdomyosarcomas, and neuroblastomas [1-3]. Infectious lymphadenitis is the most common cause of a neck mass among the acquired causes [4] and is often due to a variety of bacteria and viruses. While commonly implicated bacteria are *Staphylococcus aureus *and* Streptococcus pyogenes [5], *other infectiousagents are* *rhinovirus, influenza virus, parainfluenza virus, respiratory syncytial virus, adenovirus, reovirus, Epstein-Barr virus, herpes, coxsackie, and HIV[5]*.* Although 80-90% of masses are benign and transient, they are seldom malignant [6-8], creating anxiety in caregivers and posing a diagnostic challenge for physicians. The current research emphasizes the management of one of the most common causes of pediatric neck mass and highlights the diagnostic consideration of malignancy in children with red flags and unresolving masses with expectant therapy.

## Case presentation

A six-month-old baby boy was brought by his mother to the emergency room with a chief complaint of left-sided neck swelling for the past five days and fever for the past four days.

The boy was apparently normal prior to the illness. Initially, his mother noticed a small swelling in the left side of his neck that rapidly enlarged over the course of five days until it attained its present size. The swelling had an insidious onset and the child would cry whenever his mother touched his neck. The mother also noticed that the swelling was red with some warmth while she gave him a bath. The fever started as low grade (99 ^o^F) on the second day of swelling and then progressed to high grade (101.5-102 ^o^F) with chills and rigors. The fever responded to ibuprofen, but the child was dull during the inter-febrile period. The fever had no diurnal variation. His mother added that the swelling increased in size despite the usage of amoxicillin and clavulanic acid empirically for two days, as prescribed by her pediatrician.

There was no history of similar swellings elsewhere in the body. There was no history of trauma, rash, bleeding manifestations, night sweats, exposure to cats, or weight loss. Past medical history is unremarkable. He had no significant allergy or family history.

The infant was born through a normal vaginal delivery at term and cried immediately after birth, with a weight of six pounds. There were no significant antenatal, natal, and postnatal problems. The boy was on complementary feeds and immunized as per schedule. His development was normal in all domains.

Upon general examination, he was alert and conscious. There were no dysmorphic facies or neurocutaneous markers on his head-to-toe assessment. He weighed 17 lb, with a length of 67 cm and a head circumference of 42 cm. He was febrile (T:101.5^ o^F), with a heart rate of 120/min, blood pressure of 90/60mmHg, and respiratory rate of 30/min. He maintained 98-99% saturation on room air.

A local examination of the neck revealed a single, round swelling in the left anterior cervical area measuring 3.5 x 3 cm with irregular borders and softness in consistency. Upon palpation, the swelling was mobile and tender. It was noncompressible, nonpulsatile, and noncystic. The skin over it was warm with an area of erythema with no sinuses or fistulas. His systemic examination revealed no abnormalities.

Based on clinical presentation, a differential diagnosis of acute infectious lymphadenitis, reactive lymphadenopathy, and neoplastic lesions was considered, and an extensive workup was planned accordingly.

His initial workup on day 1 of admission included a hemogram that showed elevated white blood count (20,000/ μL) with absolute lymphocytosis (47%) and normocytic normochromic anemia on peripheral blood smear. C-reactive protein was elevated (2.5 mg/dL), with unremarkable urine analysis, monospot test, and respiratory viral PCR (Biofire RP2.1). Blood culture had no growth at 72 hours.

**Figure 1:**
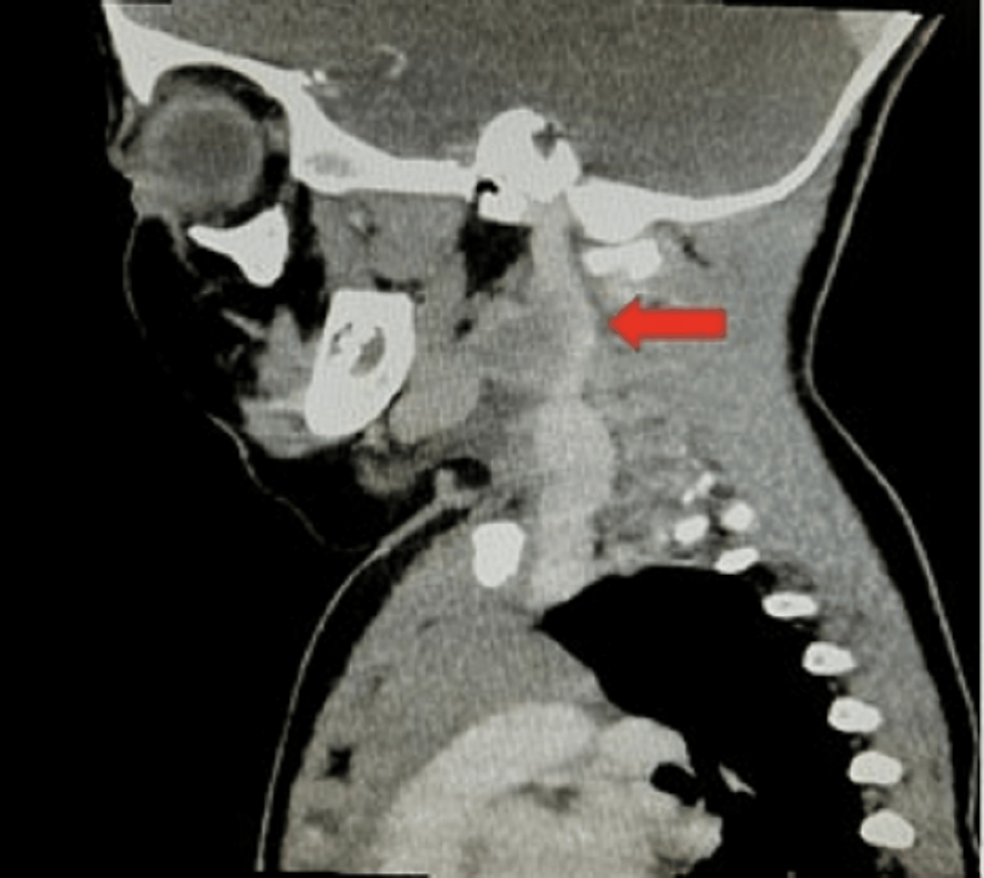
CT with contrast sagittal section showing heterogeneous attenuation on the left anterior cervical area of the neck (pointed by red arrow)

**Figure 2:**
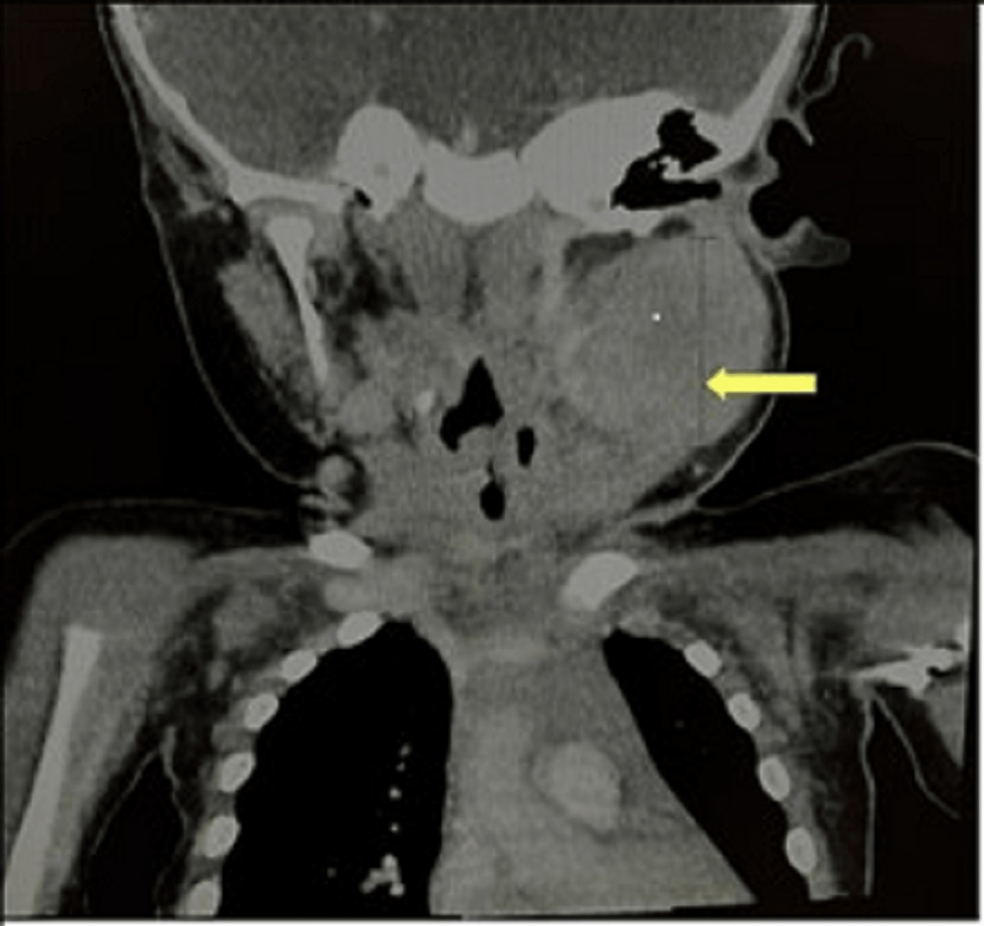
CT with contrast coronal section showing a 3.5 cm area of decreased attenuation on the left anterior cervical area (pointed by yellow arrow)

**Figure 3:**
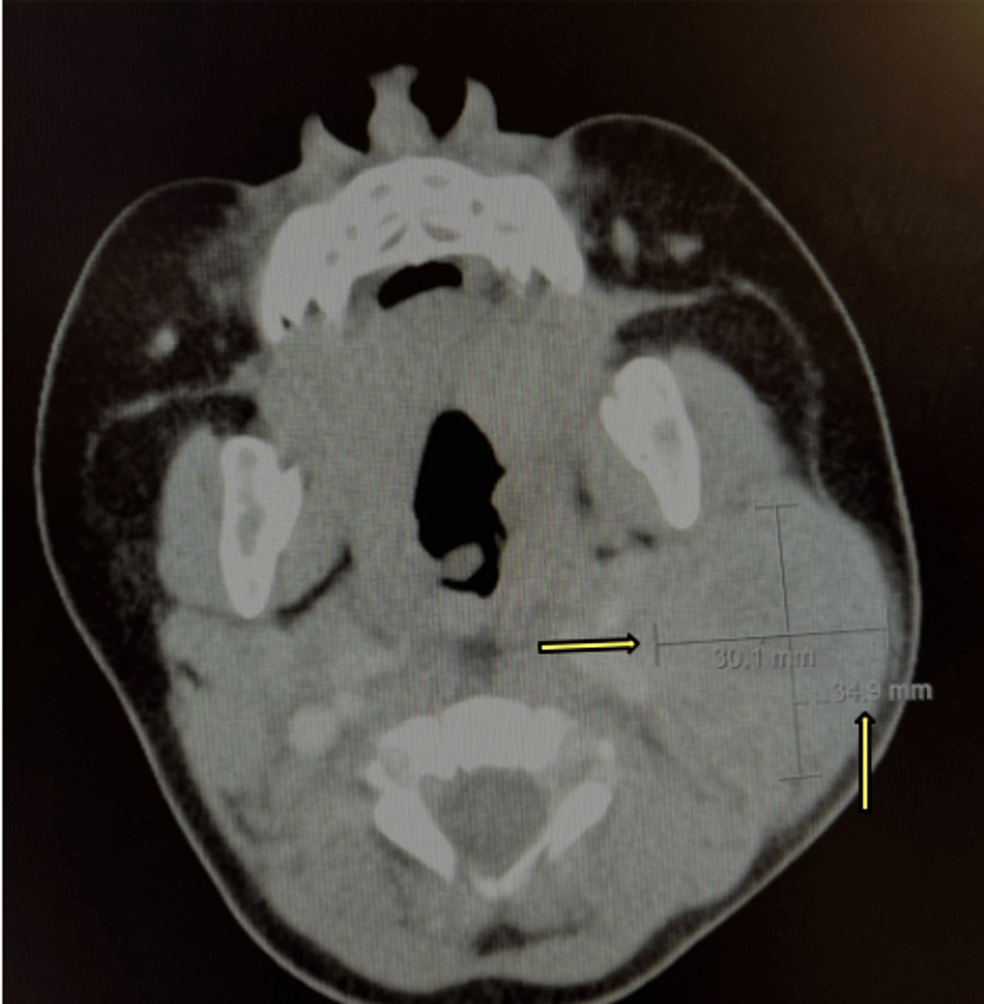
Axial CT with contrast showing heterogeneous enhancement (pointed by yellow arrows)

Computerized tomography (CT) scan of the neck with contrast showed a 3.5 x 3 x 3.5 cm heterogeneous lesion in the left anterior cervical area with a differential diagnosis of neoplasia, lymph node with possible central necrosis, and inflammation (Figures 1-3).

A diagnosis of acute cervical lymphadenitis was made, and the child was started empirically on oral azithromycin (10 mg/kg/day) and IV clindamycin (40 mg/kg/day) with supportive therapy. However, on day three of admission, the swelling size increased clinically and became more erythematous. IV ceftriaxone (50 mg/kg/day) was added to the regimen, and further diagnostic workup was planned with ultrasonogram (USG) and biopsy to rule out possible neoplasia and other uncommon infectious causes.

**Figure 4:**
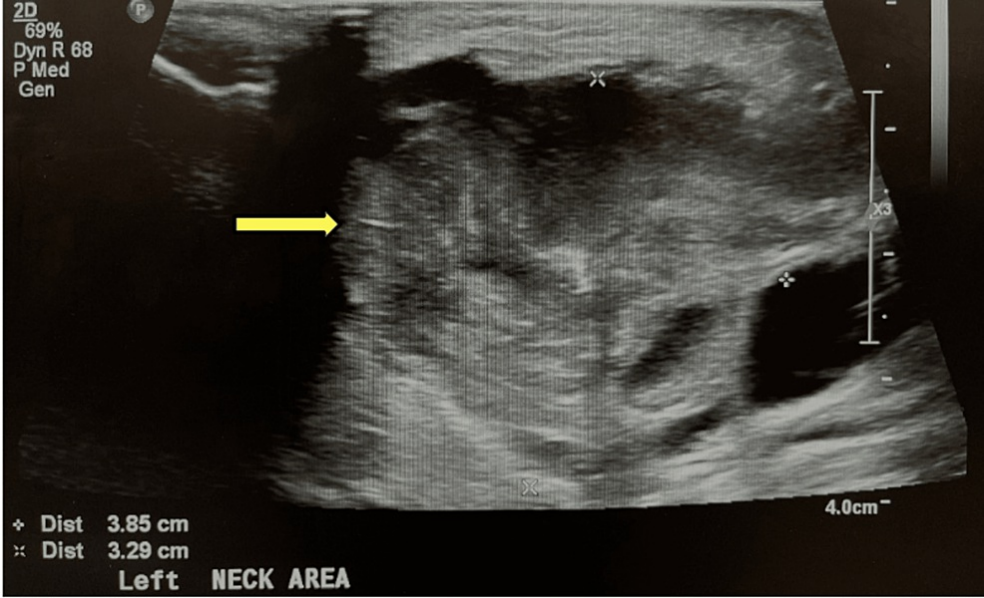
USG showing irregular heterogeneous mass in the left neck (pointed by yellow arrow)

USG measured a 39 x 33 x 28 mm irregular left-sided neck mass with heterogeneous echotexture and surrounding soft tissue swelling, consistent with CT (Figure 4).

The biopsy of the neck mass performed under anesthesia revealed necrosis, cystic degeneration, and dense, suppurative acute inflammation of fibroadipose tissue stroma, which was consistent with an organizing abscess. A wound culture obtained from the incision and drainage of the mass revealed MRSA, sensitive to clindamycin (Table 1), which was already a part of his antibiotic regimen.

**Table 1:** Antibiotic susceptibility patterns for MRSA

Drug	Interpretation	MIC
Benzylpenicillin	R	≥0.5
Clindamycin	S	≤0.25
Erythromycin	S	≤0.5
Gentamicin	S	≤0.5
Inducible clindamycin resistance	Negative	Negative
Linezolid	S	2
Oxacillin	R	≥4
Tetracycline	S	≤1
Trimethoprim/sulfamethoxazole	S	≤10
Vancomycin	S	1
MIC: Minimum inhibitory concentration; R: Resistant; S: Sensitive		

The child showed clinical improvement on day five of admission. The fever gradually subsided and swelling began to decrease in size. He was discharged with oral antibiotics for five days and put on follow-up.

## Discussion

Fourty-four percent of children aged under five with palpable lymphadenitis were reported to have a benign etiology [9]. A rapidly enlarging, unilateral, tender cervical node in a child should raise suspicion for bacterial lymphadenitis. The commonly involved nodes in bacterial lymphadenitis in declining order are the submandibular, upper cervical, submental, occipital, and lower cervical nodes [5]. *S. aureus *and* S. pyogenes* account for 40-80% of cases in the under-five pediatric population with unilateral lymphadenitis [5]. Other less-frequently implicated anaerobes are *Bacteroides, Peptostreptococcus, Propionibacterium acnes, *and *Fusobacterium* [10]. Very rarely, cases have been reported with *Francisella tularensis, Pasteurella multocida, Yersinia pestis, Haemophilus influenza *type b*, Streptococcus pneumoniae, Yersinia enterocolitica*, and *Staphylococcus epidermidis *[11].

An empiric antibiotic course should be considered in cervical lymphadenitis with systemic symptoms (e.g., fever, chills), presence of tender, erythematous nodes, and unilateral node ≥3cms. A 10-day course of oral cephalexin, amoxicillin, and clavulanic acid or clindamycin is suggested upon expert opinion, targeting common pathogens such as *Staphylococcus aureus *and *Streptococcus pyogenes* [12].

Diagnostic workup should be extended if there is no improvement with initial antibiotics. In such children, antibiotic coverage should also be extended to include strains of MRSA, congruent to our case. While most isolates in lymphadenitis are methicillin-sensitive, there has been an increased occurrence of community-acquired methicillin-resistant *S. aureus* (CA-MRSA) infections [13-16]. Evidencing the inclining trends of methicillin-resistant strains in healthy children [17,18], CA-MRSA could surpass the existing microbes causing lymphadenitis in the future.

Although most pediatric neck masses are benign and self-resolving, watchful waiting with patience is needed [12]. However, one should always give special consideration to red flags that suggest further workup for malignancy (Table 2).

**Table 2:** Red flag signs in a child with neck mass suggesting further workup for malignancy

S No.	Clinical feature
1.	Weight loss
2.	Sustained fevers/night sweats
3.	Generalized lymphadenopathy
4.	Pancytopenia
5.	Mass persisting ≥ 6 weeks
6.	Lymph node ≥ 3cms
7.	Thyroid mass
8.	Supraclavicular mass
9.	Hard, irregular mass
10.	Fixed mass

There is little evidence to determine the best approach for pediatric neck mass; however, existing algorithms are postulated based on expert opinion [19]. A biopsy is always recommended in lymph nodes that are rubbery, firm, immobile, painless, and which persist for longer than six weeks or enlarge despite appropriate antibiotic coverage [8,20]. If histological sections of the biopsy confirm neoplasia, metastatic work-up and staging are then accomplished for early therapeutic intervention.

## Conclusions

While most pediatric neck masses are benign and transient, few cases put physicians in a diagnostic dilemma. They are unresolving with expectant management and require broader antibiotic coverage targeting MRSA (similar to our case). However, an extensive workup is needed for masses that are unresolving despite appropriate antibiotic coverage and in children with red flag signs. The current case report emphasizes the management of MRSA cervical lymphadenitis and adds further evidence to its increasing trends in the pediatric population.
